# Tumor growth suppressive effect of IL-4 through p21-mediated activation of STAT6 in IL-4Rα overexpressed melanoma models

**DOI:** 10.18632/oncotarget.8111

**Published:** 2016-03-16

**Authors:** Hye Lim Lee, Mi Hee Park, Ju Kyoung Song, Yu Yeon Jung, Youngsoo Kim, Kyung Bo Kim, Dae Yeon Hwang, Do Young Yoon, Min Jong Song, Sang Bae Han, Jin Tae Hong

**Affiliations:** ^1^ College of Pharmacy and Medical Research Center, Chungbuk National University, Cheongju, Chungbuk, 361-951, Republic of Korea; ^2^ Department of Pharmaceutical Science, College of Pharmacy, University of Kentucky, Lexington, Kentucky 40536, USA; ^3^ Department of Biomaterial Science, Pusan National University, Miryang, Kyungnam, 627-706, Republic of Korea; ^4^ Department of Bioscience and Biotechnology, Bio/Molecular Informatics Center, Konkuk University, Gwangjin-gu, Seoul, 143-701, Republic of Korea; ^5^ Department of Obstetrics and Gynecology, Daejeon St. Mary's Hospital, College of Medicine, The Catholic University of Korea, Jung-gu, Daejeon, 301-723, Republic of Korea

**Keywords:** IL-4, STAT6, p21, melanoma, tumor growth

## Abstract

To evaluate the significance of interleukin 4 (IL-4) in tumor development, we compared B16F10 melanoma growth in IL-4-overespressing transgenic mice (IL-4 mice) and non-transgenic mice. In IL-4 mice, reduced tumor volume and weight were observed when compared with those of non-transgenic mice. Significant activation of DNA binding activity of STAT6, phosphorylation of STAT6 as well as IL-4, IL-4Rα and p21 expression were found in the tumor tissues of IL-4 mice compared to non-transgenic mice. Higher expression of IL-4, STAT6 and p21 in human melanoma tissue compared to normal human skin tissue was also found. Higher expression of apoptotic protein such as cleaved caspase-3, cleaved caspase-8, cleaved caspase-9, Bax, p53 and p21, but lower expression levels of survival protein such as Bcl-2 were found in the tumor of IL-4 mice. *In vitro* study, we found that overexpression of IL-4 significantly inhibited SK-MEL-28 human melanoma cell and B16F10 murine melanoma cell growth via p21-mediated activation of STAT6 pathway as well as increased expression of apoptotic cell death proteins. Moreover, p21 knockdown with siRNA abolished IL-4 induced activation of STAT6 and expression of p53 and p21 accompanied with reduced IL-4 expression as well as melanoma cell growth inhibition. Therefore, these results showed that IL-4 overexpression suppressed tumor development through p21-mediated activation of STAT6 pathways in melanoma models.

## INTRODUCTION

Interleukin-4 (IL-4) is a multifunctional cytokine that plays a critical role in the regulation of immune responses. The IL-4Rs are divided with type I IL-4R consisting IL-4Ra and the common gamma chain (γc), and type II consisting IL-4Rα and IL-13Rα1. Type I IL-4R is expressed in lymphocytes and type II IL-4R is expressed in solid tumors and non-hematopoietic cells [1]. Human tumor cell lines including expressing IL-4R has been shown to mediate anti-proliferative activity of IL-4 [2–4]. It is noteworthy that cytokines released in response to infection, inflammation and immunity can function to inhibit tumor development and progression [5]. IL-4 is a pleiotropic type cytokine produced primarily by CD4+ T cells. [6, 7]. IL-4 has an inhibitory role in angiogenesis [8], as well as in cancer growth, such as multiple monocyte [9], lung [10], kidney [11], liver [12], breast [13]. IL-4 transfection of cDNAs into mouse melanoma cells decreased the tumorigenicity of B16F10 melanoma cells by the activation of immune response [14].

A variety of important cellular functions are regulated by cytokines. The Janus kinase (JAK)-signal transducer of activators of transcription (STAT) pathway is now recognized as an evolutionarily conserved signaling pathway employed by diverse cytokines, interferons, growth factors, and related molecules [15]. Following binding of a ligand to its cognate receptor, receptor-associated Jaks are activated. STAT proteins are then in turn activated by tyrosine phosphorylation by Jak kinases, allowing their dimerization and subsequent translocation into the nucleus, where they modulate expression of target genes [16]. STAT proteins are critical mediators of cytokine signaling. Compared with normal cells and tissues, constitutively activated STATs have been detected in a wide variety of human cancer cell lines and primary tumors including a melanoma skin cancer. Numerous studies have demonstrated constitutive activation of STATs in particular STAT1, STAT3 and STAT5 by cytokines in a large number of diverse human tumor cell lines [17, 18]. STAT6 is activated by IL-4 and IL-13, and plays a predominant role in oncogenesis. IL-4 induces apoptosis of human hepatocytes through STAT6 activation, and increased caspase activation [12]. IL-13 also induces STAT6 dependent apoptosis in HT-29 colon cancer cells [19]. IL-12 activates STAT4 and enhances antitumor activity through IFN-γ production. IL-27 activates STAT1 and enhances antitumor activity [20]. However, the relationship between melanoma skin cancer and activation of STAT6 has not yet been studied.

The cyclin-dependent kinase inhibitor p21 (also known as p21^WAF1/Cip1^) promotes cell cycle arrest in response to many stimuli. It is well positioned to function as both a sensor and an effector of multiple anti-proliferative signals [21]. Expression of p21 induced apoptosis in prostate [22], skin [23], and thyroid cancer cells [24] as well as melanoma [25]. It is reported that expression of p21 is increased in human malignant melanoma tissues compared to normal tissue [26]. Expression of p21 is also associated with p53 and proliferating cell nuclear antigen (PCNA) expression level in clinical stage I cutaneous malignant melanoma patients [27]. Its expression is usually induced by p53 protein after DNA damage, and it has a role in inhibiting several cyclin dependent kinases (CDK2, CDK3, CDK4 and CDK6) resulting in G1 cell cycle arrest and block of transition in S phase [28]. p21 is also able to determine cell cycle arrest in G2 phase, through its interaction with PCNA, an essential cofactor for DNA polymerases [29]. Recently, it was reported that STATs protein and p21 protein are associated during apoptosis. STAT1 protein is essential for cell growth suppression in response to IFN-γ [30]. The STAT signaling pathway appears to negatively regulate the cell cycle by inducing CDK inhibitors p21 in response to IFN-γ in A431 human epithelial carcinoma cells [31]. Thrombin inhibits cell growth by both up-regulation of STAT1 dependent p21*waf/cip1* and induction of caspases via its PAR-1 receptor in DU145 prostate cancer cells [32]. However, effect of IL-4 on STAT6 and p21 dependent cell cycle arrest has not been studied.

Malignant melanoma is a common primary malignant cutaneous tumor derived from transformed epidermal melanocytes [33]. The incidence of cutaneous melanoma has more than doubled over the last decades making it one of the fastest rising cancers worldwide [34]. Many possible mechanisms and target therapeutics for melanoma treatment have been demonstrated but clear cut mechanisms and therapeutics have not been reported. Therefore, in the present study, to evaluate the role of IL-4 in skin tumor growth, we investigated whether overexpression of IL-4 inhibits melanoma cell and tumor growth through modulation of p21-mediated STAT6 pathways.

## RESULTS

### Effect of IL-4 on cancer cell growth and apoptotic cell death

**Figure 1:**
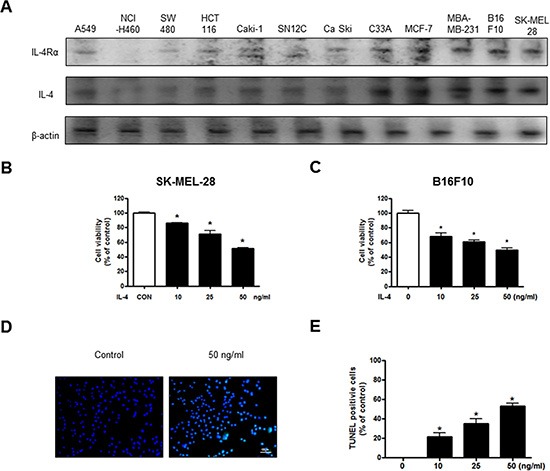
Effect of IL-4 on cancer cell growth and apoptotic cell death Expression of IL-4Rα in various cancer cell line including B16F10 melanoma cells (**A**). Concentration-dependent effect of IL-4 on the MTT viability assay in SK-MEL-28 and B16F10 after 24 hr (**B** and **C**). The B16F10 melanoma cells were treated with IL-4 for 24 hr, and then labeled with DAPI and TUNEL solution (**D**). Total number of cells in a given area was determined by using DAPI nuclear staining (fluorescent microscope). The green color in the fixed cells marks TUNEL-labeled cells. The apoptotic index was determined as the DAPI-stained TUNEL-positive cell number/total DAPI stained cell number (magnification, 200×) (**E**). Values were means ± S.D. of three experiments. *(*P* < 0.05) indicates statistically significant differences from the control cells.

The expression of IL-4Rα mediates the action of IL-4, so we performed the Western blot analysis to investigate the IL-4Rα expression of various cancer cells (Figure 1A). The highest expression of IL-4 and IL-4Rα was found in melanoma cells among the cancer cells (Figure 1A). We then analyzed cell growth by MTT assay. A549, NCI-H460, SW 480, HCT 116, Caki-1, SN12C, Ca Ski, C33A, MCF-7 and MDA-MB 231 cells were treated with IL-4 (50 ng/ml) for 24 hr. IL-4 inhibited cell growth of these cancer cells, and the most inhibition was found in melanoma (Supplementary Figure 1). The growth inhibition was associated with IL-4 and IL-4Rα expression (Figure 1A). Since IL-4 and IL-4Rα were highly expressed in melanoma cells, and melanoma cell growth has been known to be significantly affected by cytokines [35], we chose melanoma cells for further mechanism and *in vivo* studies to investigate the role of IL-4 in melanoma growth. SK-MEL-28 and B16F10 cells were treated with several concentrations of IL-4 (10, 25 and 50 ng/ml) for 24 hr, and found cell growth inhibition in a dose dependent manner (Figure 1B, 1C). We then performed DAPI staining followed by TUNEL staining assays. The double labeled cells were analyzed by a fluorescence microscope to determine that the inhibition of cell growth by IL-4 was due to the induction of apoptotic cell death. Reversely, consistent with cell growth inhibitor effects, apoptotic cell death was significantly increased in rhIL-4 treated B16F10 melanoma cells, respectively. The number of apoptotic cells (DAPI-positive TUNEL-stained cells) in B16F10 melanoma cell culture was increased to about 58% of cells, respectively, at a concentration of 50 ng/ml (Figure 1D, 1E).

### Effect of IL-4 on expression apoptotic cell death regulation proteins

**Figure 2:**
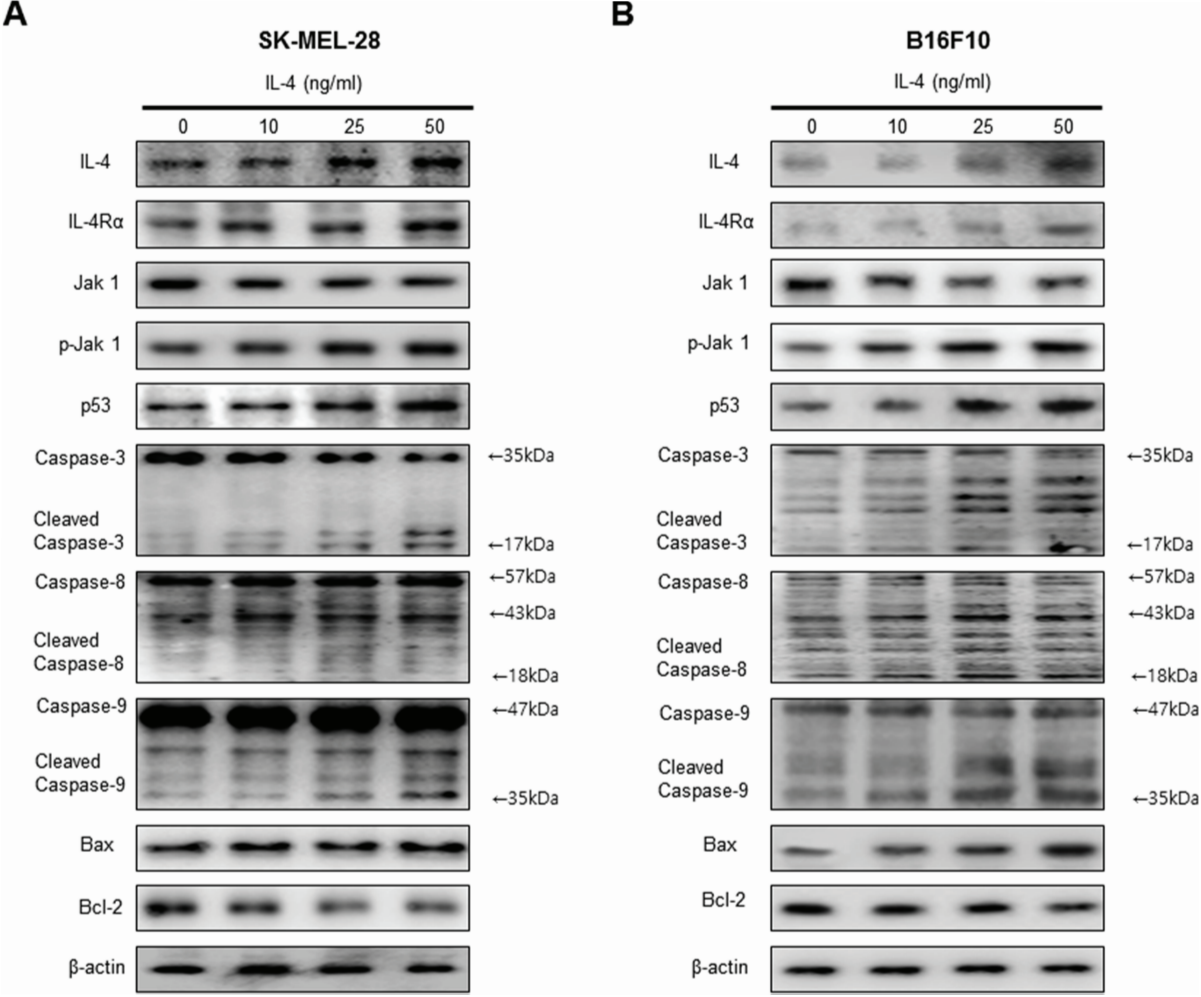
Effect of IL-4 on expression of apoptotic cell death regulation proteins Western blot analysis with the antibodies against IL-4, IL-4Rα, Jak1 and p-Jak1 and apoptosis regulatory proteins including p53, Caspase-3, caspase-8, caspase-9, Bax, Bcl-2 and β-actin (**A** and **B**). β-actin protein was used an internal control. Each band is representative for three experiments.

The activation of cell death regulatory proteins including caspases-3, -8 and -9 as well as Bax, leads to apoptosis in cancer cells. To determine the association of the expression of cell death regulatory proteins and IL-4, the expression of apoptotic proteins was investigated by Western blots. IL-4 treatment clearly increased IL-4 and IL-4Rα expression. Consistent with increased IL-4, and IL-4Rα expression, the expression of pro-apoptotic proteins; Bax and cleaved form of caspase-3, -8, -9 were increased by the treatment of IL-4. However, the expression of Bcl2 was decreased by the treatment of IL-4 in SK-MEL-28 (Figure 2A) and B16F10 melanoma cells (Figure 2B).

### Effect of IL-4 on STAT6 activation and expression of p21

**Figure 3:**
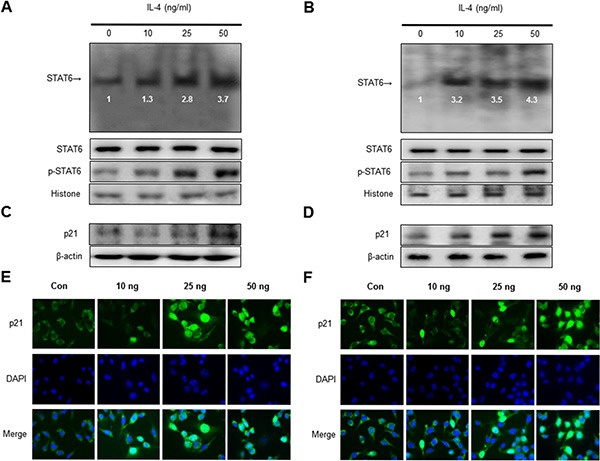
Effect of IL-4 on STAT6 activation and expression of p21 Nuclear extract from melanoma cells treated with IL-4 (10, 25, and 50 ng/ml) for 1 hr was incubated in binding interaction of 32^P^-end-labeled oligonucleotide containing the STAT6 sequence. The present EMSA results are representatives of three experiments. The cells treated with IL-4 for 1 hr was incubated and were lysed, nuclear proteins were used to determine the expression of STAT6, p-STAT6 and Histone (internal control) in SK-MEL-28 human melanoma cells and B16F10 murine melanoma cells (**A** and **B**). Western blot analysis with antibodies against p21 and β-actin (**C** and **D**). β-actin protein was used an internal control. Each band is representative for three experiments. Melanoma cells were treated with 10 to 50 ng/mL IL-4 for 24 h, and then the intracellular location of p21 was determined by immunofluorescence confocal scanning microscope (magnification, 630×). Double staining (Merge) with p21 and 4′, 6-diamidino-2-phenylindole (DAPI) staining shows the localization of p21 in the nucleus (**E** and **F**).

To investigate whether IL-4 activates STAT6 activation, an EMSA for detecting DNA binding activity of STAT6 was carried out. We found that IL-4 untreated SK-MEL-28 and B16F10 melanoma cells showed lower constituted activation of STAT6 in SK-MEL-28 and B16F10 melanoma cells. However, the treatment IL-4 dose dependently increased DNA binding activity of STAT6. Agreed with the increment of DNA binding activity of STAT6, the phosphorylation of STAT6 in nuclei was increased by IL-4 treatment in SK-MEL-28 (Figure 3A) and B16F10 melanoma cells (Figure 3B). The band of STAT6 was supershifted by STAT6 specific antibody in SK-MEL-28 melanoma cells (Supplementary Figure 2). Expression of p21 is associated with apoptotic cell death and growth arrest of cancer cells in conjunction with STAT6 activity. Therefore, to investigate expression of p21 in SK-MEL-28 human melanoma cell and B16F10 murine melanoma cell undergoing apoptotic cell death, we performed a Western blot analysis. IL-4 treatment clearly increased expression of p21 in a dose dependent manner (Figure 3C, 3D). We also demonstrated p21 activity by an immunofluorescence analysis by a confocal microscope. SK-MEL-28 and B16F10 cells were treated with IL-4 (0–50 ng/ml) for 24 h. Translocation of p21 into the nucleus was also increased significantly in a dose dependent manner (Figure 3E, 3F).

### Reversed effect of p21 siRNA on IL-4-induced cell growth inhibition and STAT6 activation

**Figure 4:**
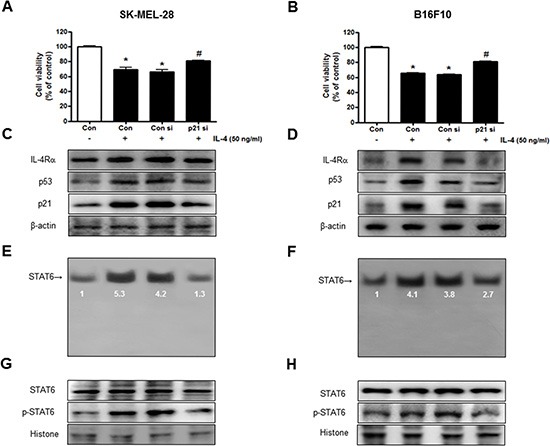
Reversed effect of p21 siRNA on IL-4-induced cell growth inhibition and STAT6 activation The melanoma cells were transfected with the p21 siRNA (100 nM) for 24 hr, the cells were then treated with IL-4 (50 ng/ml) for 24 hr. After treatment, cell viability was measured by MTT assay (**A** and **B**), and the expression of apoptosis regulatory proteins (**C** and **D**) and STAT6 activity (**E** and **F**) were determined as described above. Cell growths are means ± S.D. of three experiments. *(*P* < 0.05) indicates statistically significant differences from control group. # (*P* < 0.05) indicates statically significant differences from rhIL-4 treated group.

To determine the relationship between p21 expression and SK-MEL-28 and B16F10 melanoma cells growth and STAT6 activity in the inhibitory effects of IL-4, we transfected SK-MEL-28 and B16F10 melanoma cells with p21 siRNA using a transfection agent. The melanoma cells were transfected with 100 nM siRNA of p21 for 24 hr, and then treated with IL-4 (50 ng/ml) for 24 hr. Knock down of p21 almost completely reversed the cell growth inhibitory effect of IL-4 in SK-MEL-28 (Figure 4A) and B16F10 cells (Figure 4B). We also found that expression of IL-4, IL-4Rα, p53 and p21 was also reversed (Figure 4C, 4D). IL-4-increased activity of STAT6 by IL-4 was also abolished by transfection with p21 siRNA in SK-MEL-28 and B16F10 melanoma cells (Figure 4E, 4F). Increased nucleus phosphorylation of STAT6 by IL-4 was also abolished by transfection with p21 siRNA in SK-MEL-28 and B16F10 melanoma cells (Figure 4G, 4H).

### IL-4 inhibited tumor growth in melanoma model

**Figure 5:**
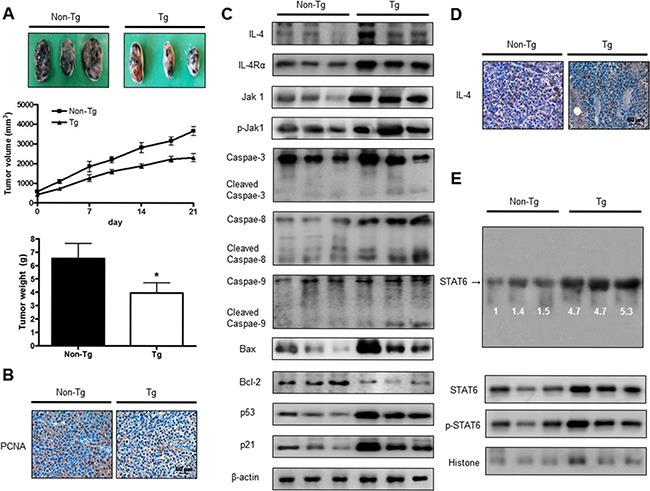
IL-4 inhibited tumor growth in melanoma model Tumor growth inhibition (as assessed by tumor volume) in B16F10 bearing IL-4 mice. Tumor burden was measured twice per week using a caliper, and volume was calculated with volume length (mm) × width (mm) × height (mm)/2. Tumor weight and volume are presented as means ± S.D. (**A**). Immunohistochemistry was used to determine expression levels of PCNA in tissues of IL-4 mice and non-transgenic mice (**B**). The expression of IL-4 and apoptotic proteins was detected by western blotting using specific antibodies; IL-4, IL-4Rα, Jak1, p-Jak1, cleaved caspase-3, Bcl-2 and β-actin (**C**). β-actin protein was used an internal control. Tumor sections were analyzed by immunohistochemistry for detection of IL-4 expression in tumor tissues (**D**). STAT6 activity in tumor tissues was detected by EMSA and nuclear location of STAT6 was determined by western blotting. (**E**). All values represent mean ± SD from five animal tumor sections. *(*P* < 0.05) indicates significantly different from the control group.

To identify the effect of IL-4 on tumor growth, we measured the tumor growth in a melanoma model. We conducted *in vivo* study after confirmation higher expression of IL-4 in serum of Luc/IL-4/CNS-1mice compared to Non-Tg mice (Supplementary Figure 3). The results revealed that tumor volume and weight in B16F10 bearing IL-4 mice were much lower compared to non- transgenic mice. Tumor growth in IL-4 mice was less than the tumor growth in non-transgenic mice (Figure 5A). Expression of PCNA was decreased in IL-4 mice (Figure 5B). In the B16F10 bearing IL-4 mice group, expressions of IL-4, IL-4Rα, phosphorylation of Jak1 and pro-apoptotic proteins including cleavage caspase-3 were concomitantly increased (Figure 5C). Immunohistochemical analysis showed that the number of IL-4 immunoreactive cells was higher in the tumor tissues of IL-4 mice (Figure 5D). We also found higher binding activity of STAT6 in B16F10 bearing IL-4 mice compared to non-transgenic mice. Agreed with the increment of STAT6 and cytosolic phosphorylation of STAT6, as well as the nucleus phosphorylation of STAT6, were increased (Figure 5E).

### Expression of IL-4, STAT6 and p21 in human melanoma patient tissue

**Figure 6:**
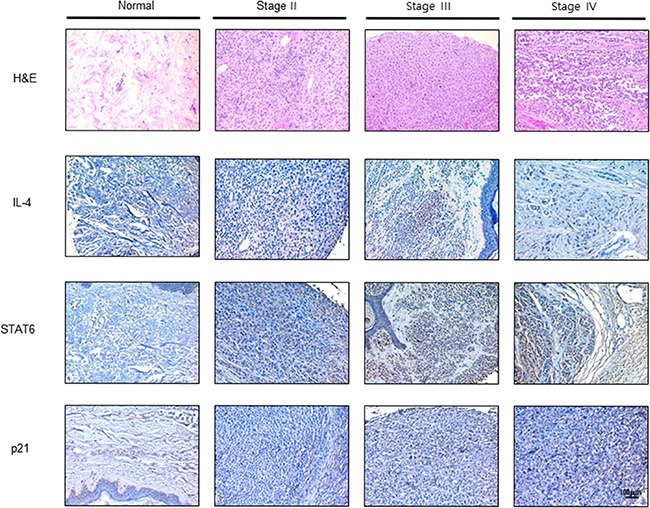
Expression of IL-4, STAT6 and p21 in human melanoma patients Human normal skin or melanoma tissue sections (Stage II–IV) were processed and stained with H & E and immunohistochemistry analysis of the expression of IL-4, STAT6 and p21. The figures represented 3 samples of each cancer stage.

We analyzed expression of IL-4, STAT6 and p21 in human melanoma tissues (Stage II–IV) and normal tissues by immunohistochemistry analysis. IL-4, STAT6 and p21 are concomitantly and progressively expressed in human melanoma tissues in a cancer stage dependent manner (Figure 6).

## DISCUSSION

Potential anti-melanoma drugs, including anti-angiogenic agents, kinase inhibitors, non-antisense oligonucleotides and proteasome inhibitors (PI) are currently in clinical trials. It has been proposed that the resistance of metastatic melanoma to traditional chemotherapeutic and new anticancer agents is mainly due to the increased resistance of melanoma cells to drug-induced apoptosis [36]. Metastatic melanoma patients are often treated with one of several targeted therapies approved by the Food and Drug Administration (FDA), including vemurafenib (Zelboraf^®^), dabrafenib (Tafinlar^®^) and trametinib (Mekinist^®^). Recently, pembrolizumab has been contingently approved for the treatment of patients with advanced melanoma based on their durable response, high response rate, and favorable safety profile [37]. Pembrolizumab is a highly selective humanized monoclonal IgG4 antibody directed against the programmed death-1 (PD-1) receptor on the cell surface [38]. PIs (bortezomib, ALLN, MG-132 and epoxomicin) triggered apoptosis in melanoma cell lines accompanied by cytochrome C release, activation of multiple caspases and by significant increase in number of treated melanoma cells in sub-G1 phase of cell cycle [39]. Although these therapeutic treatments have achieved significant benefits for patients in clinical trials, its effectiveness and administration have been limited by toxic side effects. The most frequent side effect (incidence > 30%) associated with bortezomib in clinical trials include asthenic conditions, gastrointestinal events, hematological toxicity, peripheral neuropathy characterized by decreased sensation, paresthesia and a high rate of shingles [40, 41]. Spontaneous regressions of malignant melanoma lesions have been observed via activated lymphocytes [42]. Indeed, infiltration of T cells within melanoma tumors can be associated with a better prognosis [43]. Thus, modulation of immune responses by the use of recombinant cytokines or cytokine is one of the strategies for cancer therapy to reduce side effects [44].

In the present study, we found that IL-4 overexpression inhibited cell growth of cultured SK-MEL-28 human melanoma cells and B16F10 murine melanoma cells *in vivo* through p21-mediated activation of STAT6. IL-4 has an inhibitory role in angiogenesis [8], as well as in cancer growth such as multiple monocyte [9], lung [10], kidney [11], liver [12], and breast [13] cancer cells. Human tumor cell lines expressing IL-4R has been shown to mediate anti-proliferative activity of IL-4 [2, 4, 45]. To see whether IL-4R expression could be related to an anti-cancer effect of IL-4, we investigated the IL-4R expression pattern in several cancer cell lines. Expression of IL-4Rα on melanoma cell lines was higher than other cancer cell lines, which was associated with higher cancer cell growth inhibition and apoptotic cell growth. IL-4 down-regulated the surface expression of IL-4Rα on one human renal cancer cells, gastric cancer cells and breast cancer cells [46–48], and thus showed much higher cell growth-inhibitory effects of IL-4 than IL-4R non expressed cells [46–48]. Other cytokine receptors have been shown to mediate the anti-cancer effect of their cytokines. IL-13Ra2 has a critical role to mediate the anti-tumor effect of IL-13 in breast cancer models [49]. IL-15Rα also mediates the anti-tumor effect of IL-15 in a B16 melanoma mouse model. [50]. IL-12R triggers the anti-tumor effect of IL-12 in ovarian cancer [51]. These data suggest that IL-4 could suppress melanoma cell growth via receptor-mediated cell death effect.

Activation of the p21 pathway of growth arrest and apoptosis is a well known signaling pathway in the regulation of melanoma cell growth. Keratinocyte derived from p21−/− mice results in a markedly increased susceptibility to 7, 12-dimethylbenzanthracene (DMBA) induced skin carcinoma formation [52]. Moreover, p21 are concomitantly and progressively expressed in human melanoma tissues in a cancer stage dependent manner. It is also reported that expression of p21 in human tumor tissue is increased in gastric [53], laryngeal [54] cancer cells as well as melanoma [26]. It is reported that all trans retinoic acid (atRA) induce apoptosis in WM164 primary melanoma cells and 451Lu metastatic melanoma cells. Expression of p53, p21 and bax was increased, and bcl-2 was decreased in melanoma cells after exposure to atRA at different concentrations for various periods of time [25]. Thus, the cyclin-dependent kinase inhibitor p21 promotes cell cycle arrest in response to many stimuli. It is well positioned to function as both a sensor and an effector of multiple anti-proliferative signals. In our data, inhibition of melanoma cell growth by rhIL-4 accompanied increased expression of p21 in a dose dependent manner. But, inhibition of cell growth by higher expression of p21 response to IL-4 was abolished by transfection with p21 siRNA in SK-MEL-28 and B16F10 melanoma cells. Expression of p21 was also increased in the tumor of IL-4 mice compared with those of non-transgenic mice. Cell-cycle regulation in mammalian cells is affected by the ordered activation of a group of related enzymes known as the cyclin-dependent kinase (CDK) [55]. p21 as a CDK inhibitor is a key cell cycle regulator that arrests cells in the G1 and G2 phases [56]. Our present results showed that expression of IL-4 and IL-4Rα in SK-MEL-28 and B16F10 melanoma cells were increased. However, treatment of p21 siRNA in SK-MEL-28 and B16F10 reversed IL-4-induced melanoma cancer cell growth inhibition. It was reported that many cytokines arrest cancer cell cycle through activation of p21 in 5637 and T-24 bladder cancer cells [57]. IFN-α increases the expression of p21 in a carcinoid tumor cell line, and thus decreases G1- and G2-phase cells, but increases S-phase population. In addition, IFN-α inhibited cyclin dependent kinases (CDK), CDK2-, CDK3-, CDK4-, and cyclin E- but not cyclin A-associated kinase activities and induced cell cycle arrest in carcinoid tumor [58]. Thus, our data suggests that expression of p21 is associated with the tumor growth suppressive effect of IL-4.

STAT6 proteins that relay signals from activated cytokine in the plasma membrane to the nucleus, where they regulate gene transcription in the control of cancer growth [59]. So, it alone can induce apoptosis. Overexpression of mSTAT6 alone inhibited proliferation, increased the basal rate of apoptosis, and inhibited focus formation of MCF-7 human breast cancer cells [60]. STAT6 blockade by STAT6 siRNA and by anti-IL-4 inhibited IL-4-mediated apoptosis in HepG2 cells and in human hepatocytes [12]. It was reported that activation of STAT proteins is generally associated with tumor tissues. But, STAT1 expression is lower in head and neck tumor than normal tissue [61]. STAT3 is increased in gastric [62] and breast tumor [63]. STAT4 is increased in prostate tumor [64]. STAT6 is also increased in colon [65], glioblastoma [66], breast [67], prostate tumor [64] and melanoma compared to normal tissue. Cytokines have antitumor effects through STAT mediated signaling [68]. IL-12 activates STAT4 and enhances antitumor activity through IFN-production [69]. IL-27 induced activation of STAT1 enhances antitumor activity in human epidermal keratinocytes [70]. Overexpressed IL-23 enhances antitumor activity in SU-DHL-4 human B cell lymphoma cell inoculated xenograft model [71], however, it is also regulated that endogenous IL-23 promotes pro-tumor activity through STAT3 activation by inducing inflammatory responses including IL-17 production [72]. It is also reported that IL-13 involved in the IL-4R-STAT6 pathway, are necessary for tumor promotion in 15-12RM sarcoma model. Our data demonstrated that consistent with cancer cell growth inhibition, IL-4 induced DNA binding activity of STAT6 and nucleus phosphorylation of STAT6 were increased in a B16F10 melanoma cell and melanoma tumor model. We also found higher expression of p21 as well as STAT6 in human melanoma tissues compared to normal skin tissues. It is also reported that STAT proteins are related to p21-mediated apoptosis pathway. Thrombin inhibits DU145 prostate cancer cell, MEF murine fibroblast cell and CHRF human megakaryocyte cell growth via up-regulation of p21 and capases via a p53 independent and STAT1 dependent pathway [32]. IFN-γ induced up-regulation of p21 and activation of STAT1 protein in epithelial cell carcinoma [73] and ovarian cancer cell lines [74]. IL-13/4 induces p21 expression in human monocytes by an IL-13R/JAK pathway, through increased p21 gene transcription, which is probably sustained by the STAT responsive element, and that the activation of PPARγ by this cytokine can counteract this induction [75]. Cancer cell growth-inhibitory effect of STAT6 was shown to be mediated by induction of the G1 cyclin-dependent kinase inhibitors p21^Cip1/WAF1^ and p27^Kip1^ in human breast cancer cells. STAT6 knockdown resulted in enhanced cell proliferation and a decrease in p21 and p27 mRNA levels in the steroid-responsive and non-responsive T-47D and MDA-MB-231 cell lines, respectively [76]. Moreover, we found that the increment of STAT6 by IL-4 was also abolished by the transfection with p21 siRNA both in SK-MEL-28 and B16F10 cells. These data suggest that the activation of STAT6 by IL-4 is mediated by p21 expression. Thus, our data suggest that IL-4 suppresses tumor growth through p21-mediated activation of STAT6 in melanoma tumor of tissues.

## MATERIALS AND METHODS

### Materials

Recombinant human IL-4 and mouse IL-4 were purchased from R & D system (*Minneapolis*, *Minnesota*).

### Cell culture

SK-MEL-28 human melanoma cells were obtained from the Korean Cell Line Bank (Seoul, Korea). B16F10 mouse melanoma cells, A549 and NCI-H460 human lung cancer cells, SW480 and HCT116 human colon cancer cells, Caki-1 and SN12C human renal cancer cells, Ca Ski and C33A human cervical cancer cells, and MCF-7 and MDA-MB 231 human breast cancer cells were obtained from the American Type Culture Collection (Cryosite, Lane Cove NSW, Australia). A549, NCI-H460 and SW480 cells were grown in RPMI1640 (Gibco, Life Technologies, Grand Island, NY) with 10% fetal bovine serum, 100 U/ml penicillin and 100 μg/ml streptomycin at 37°C in 5% CO_2_ humidified atmosphere. B16F10, Caki-1, SN12C, Ca Ski, C33A and MCF-7 cells were grown in DMEM (Gibco, Life Technologies, Grand Island, NY) with 10% fetal bovine serum, 100 U/ml penicillin and 100 μg/ml streptomycin at 37°C in 5% CO_2_ humidified atmosphere. These cell lines were authenticated by monitoring of cell morphology, contamination inspection.

### Human samples

The human melanoma tissues and human normal skin tissues samples were purchased from US Biomax, Inc. cancer tissue bank collection (US Biomax, Inc., MD, USA).

### Cell viability assay

Cells were plated in 96-well plates and subsequently treated with IL-4 (0–50 ηg/mL) for 24 hr. After treatment, cell growth was measured by MTT [3-(4, 5-dimethylthiazol-2-yl)-2, 5-diphenyltetrazolium Bromide] assay (Sigma Aldrich, St. Louis, MO) according to the manufacturer's instructions. Briefly, MTT (5 mg/ml) was added and plates were incubated at 37°C for 2 hr before 100 μl dimethyl sulfoxide (DMSO) was added to each well. Finally, the absorbance of each well was read at a wavelength of 540 nm using a microplate reader.

### Evaluation of apoptotic cell death

TUNEL assay was performed by using the DeadEnd^TM^ Fluorometric TUNEL System (Promega, Madison, Wisconsin, USA) for *in situ* detection of apoptotic cells, according to the manufacturer's instructions. B16F10 cells (1 × 10^4^ cells/well) were cultured on 8-chamber slides, after cells were treated with IL-4. The cells and tumor tissues were washed with PBS and fixed by incubation in 4% paraformaldehyde in PBS for 1 hr at room temperature. Membrane was permeabilized by exposure to 0.1% Triton X-100 in PBS for 5 min at room temperature. For DAPI staining, slides were incubated for 15 min at room temperature in the dark with a mounting medium for fluorescence containing DAPI (Vector Laboratories, Inc., Burlingame, CA). The cells were then observed through a fluorescence microscope (Leica Microsystems AG, Wetzlar, Germany). The total number of cells in a given area was determined by using DAPI and TUNEL staining. The apoptotic index was determined as the number of DAPI-stained TUNEL-positive cells divided by the total number of cells counted × 100.

### Western blotting

SK-MEL-28 cells and B16F10 cells treated with IL-4 (0–50 ng/ml) for 24 hr were homogenized with a protein extraction solution (PRO-PREP^™^, Intron Biotechnology), and lysed by 60 min incubation on ice. The cell lysate was centrifuged at 15,000 rpm for 15 min at 4°C. Equal amounts of protein (20 μg) were separated on a SDS/12%-polyacrylamide gel, and then transferred to a polyvinylidene difluoride (PVDF) membrane (GE Water and Process Technologies, Trevose, PA, USA). Blots were blocked for 1 h at room temperature with 5% (w/v) skim milk in Tris-Buffered Saline Tween-20 [TBST: 10 mM Tris (pH 8.0) and 150 mM NaCl solution containing 0.05% Tween-20]. After a short washing in TBST, the membranes were immunoblotted with the following primary antibodies: caspase-3, caspase-9, caspase-8, Bcl-2, p53 (1:1,000 dilutions; Cell Signaling, Beverly, MA) and IL-4, IL-4Rα, JAK1, p-JAK1, STAT6, p-STAT6, Bax, p21, (1:1,000 dilutions; Santa Cruz Biotechnology, Santa Cruz, CA). The blots were performed using specific antibodies followed by second antibodies and visualization by a chemiluminescence (ECL) detection system.

### Electro mobility shift assay

The DNA binding activity of STAT6 was determined using an electrophoretic mobility shift assay (EMSA) performed as according to the manufacturer's recommendations (Promega). In short, SK-MEL-28 and B16F10 cells were cultured on 100-mm culture dishes. After treatment with IL-4 (10, 25, 50 ng/ml) for 1 hr, the cells were washed twice with PBS, followed by the addition of 1 ml of PBS, and the cells were scraped into a cold Eppendorf tube. Nuclear extracts were prepared and processed for EMSA as previously described. The relative densities of the DNA–protein binding bands were scanned by densitometry using MyImage (SLB), and quantified by Labworks 4.0 software (UVP, Inc., Upland, CA).

### Transfection of siRNA

Human and murine melanoma cells (1 × 10^4^ cells/well) were plated in 96-well plates and transiently transfected with siRNA, using a mixture of siRNA and the WellFect-EX PLUS reagent in OPTI-MEN, according to the manufacturer's specification (WelGENE, Seoul, Korea). The transfected cells were treated with 50 ng/ml IL-4 for 24 or 1 hr and then used for detecting cell viability and protein expression (1 hr culture) and STAT6 and activation (1 hr culture).

### Ethics statement

All animal experiments were approved and carried out according to the Guide for the Care and Use of Animals [Chungbuk National University Animal Care Committee, Korea (CBNU-278-11-10)].

### Animals

*pIL/Luc/CNS-1* was constructed by cloning a human IL-4 promoter with CNS-1 enhancer into *pTransLucent* (Panomics, CA) harboring the firefly luciferase gene. The *pIL/Luc/CNS-1* plasmid was microinjected into the male pronuclei of fertilized embryos obtained by crossing C57BL/6 (female) mice with DBA/2 (male) mice. The injected eggs were then transferred into the oviducts of a female pseudopregnant HR-1 recipient. Large numbers of IL-4/Luc/CNS-1 Tg mice were produced by mating IL-4/Luc/CNS-1 Tg mice and HR-1 mice [77]. The transgene was identified via DNA-PCR analysis of the genomic DNA isolated from the tails of the 3-week-old founder mice as described in elsewhere [78]. The 8 to 28 week-old IL-4/Luc/CNS-1 Tg mice (IL-4 mice) used in this study were kindly provided from the National Institute of Food and Drug Safety Evaluation of the Korea FDA (Osong, Korea). *HR1* mice were *purchased* from the *Central Lab Animal*, Inc. (Seoul, Korea). The mice were housed and bred under specific pathogen free conditions at the Laboratory Animal Research Center of Chungbuk National University, Korea.

### Experimental design

Eight-week-old IL-4 mice (*n* = 10) and eight-week-old HR-1 non- transgenic mice (*n* = 10) were injected subcutaneously with B16F10 melanoma cells (1 × 10^6^ tumor cells in 0.1 ml phosphate-buffered saline (PBS) per animal). Tumor volumes were estimated by the formula: length (mm) × width (mm) × height (mm)/2 at the end of experiment.

### Immunohistochemistry

All tissues were fixed in 4% paraformaldehyde and cut into 4 μm sections using a freezing microtome (Thermo Scientific, Germany). The sections were stained with hematoxylin and eosin (H & E) for pathological examination. For immunohistological staining, tumor sections were incubated in primary antibody. After being rinsed in PBS, the sections were subject to incubation in a biotinylated secondary antibody. The tissues were incubated for 1 hr in an avidin–peroxidase complex (ABC, Vector Laboratories, Inc., Burlingame, CA). After washing in PBS, the immunocomplex was visualized using 3, 3-diaminobenzidine solution (2 mg/10 ml) containing 0.08% hydrogen peroxide in PBS. Sections were dehydrated in a series of graded alcohols, cleared in xylene, and coverslipped using Permount (Fisher Scientific, Suwanee, GA).

### Statistical analysis

The data were analyzed using the GraphPad Prism 4 ver. 4.03 software (Graph-Pad Software, La Jolla, CA). Data are presented as mean ± SD. The differences in all data were assessed by one-way analysis of variance (ANOVA). When the *p* value in the ANOVA test indicated statistical significance, the differences were assessed by the Dunnett's test. A value of *P* ≤ 0.05 was considered to be statistically significant.

## SUPPLEMENTARY MATERIALS FIGURES


