# Correction: Tumor growth suppressive effect of IL-4 through p21-mediated activation of STAT6 in IL-4Rα overexpressed melanoma models

**DOI:** 10.18632/oncotarget.18561

**Published:** 2017-06-19

**Authors:** Hye Lim Lee, Mi Hee Park, Ju Kyoung Song, Yu Yeon Jung, Youngsoo Kim, Kyung Bo Kim, Dae Yeon Hwang, Do Young Yoon, Min Jong Song, Sang Bae Han, Jin Tae Hong

**Present:** Figure 1A contains incorrect data regarding protein expression in the cell lines.

**Correct:** The proper figure appears below.

Original article: Oncotarget. 2016; 7:23425-23438. doi: 10.18632/oncotarget.8111

**Figure 1:**
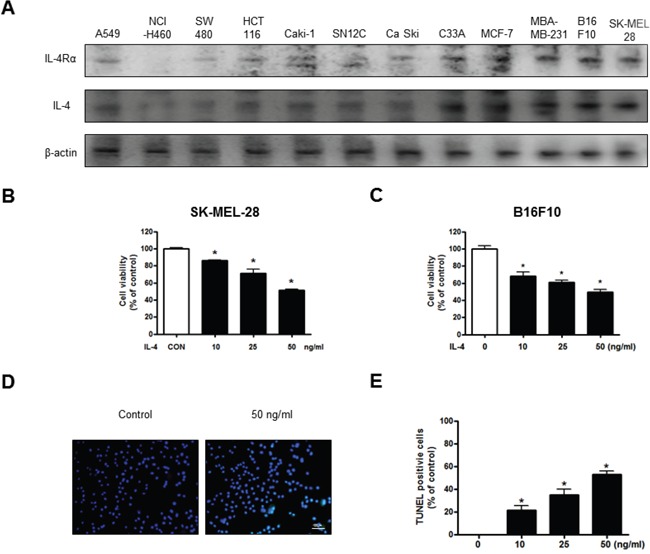
Effect of IL-4 on cancer cell growth and apoptotic cell death Expression of IL-4Rα in various cancer cell line including B16F10 melanoma cells (**A**). Concentration-dependent effect of IL-4 on the MTT viability assay in SK-MEL-28 and B16F10 after 24 hr (**B** and **C**). The B16F10 melanoma cells were treated with IL-4 for 24 hr, and then labeled with DAPI and TUNEL solution (**D**). Total number of cells in a given area was determined by using DAPI nuclear staining (fluorescent microscope). The green color in the fixed cells marks TUNEL-labeled cells. The apoptotic index was determined as the DAPI-stained TUNEL-positive cell number/total DAPI stained cell number (magnification, 200×) (**E**). Values were means ± S.D. of three experiments. *(*P* < 0.05) indicates statistically significant differences from the control cells.

