# Supplementary material for: Tumor growth suppressive effect of IL-4 through p21-mediated activation of STAT6 in IL-4Rα overexpressed melanoma models
Source: Oncotarget. 2016 Mar 16;7(17):23425–38. doi: 10.18632/oncotarget.8111 (PMC5029637; doi:10.18632/oncotarget.8111)
Supplement: Supplementary file 1 [file oncotarget-07-23425-s001.pdf]

# Tumor growth suppressive effect of IL-4 through p21-mediated activation of STAT6 in IL-4Rα overexpressed melanoma models

## Supplementary Materials

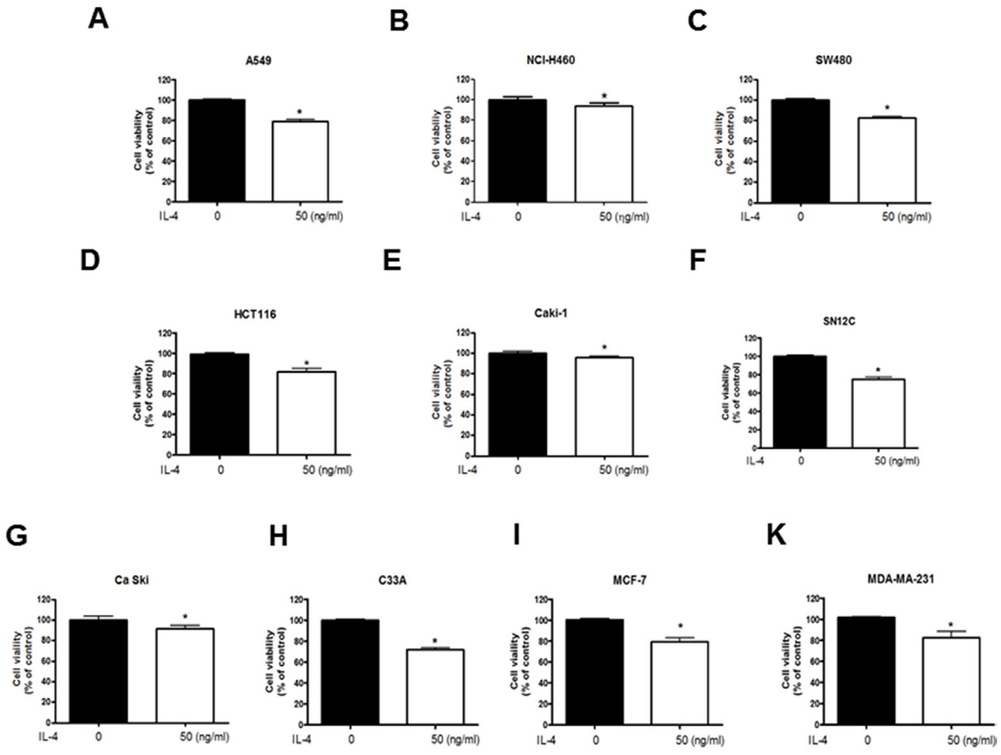


**Supplementary Figure S1: Effect of rhIL-4 on cell viability of various cancer cells.** Concentration-dependent effect of rhIL-4 on the several cancer cell growth; lung (**A** and **B**), colon (**C** and **D**) and renal (**E** and **F**) and cervical (**G** and **H**) and breast (**I** and **J**) after 24 hr treatment. The data were expressed as the mean ± S.D. of three experiments. *($P < 0.05$) indicates statistically significant differences from the control group.

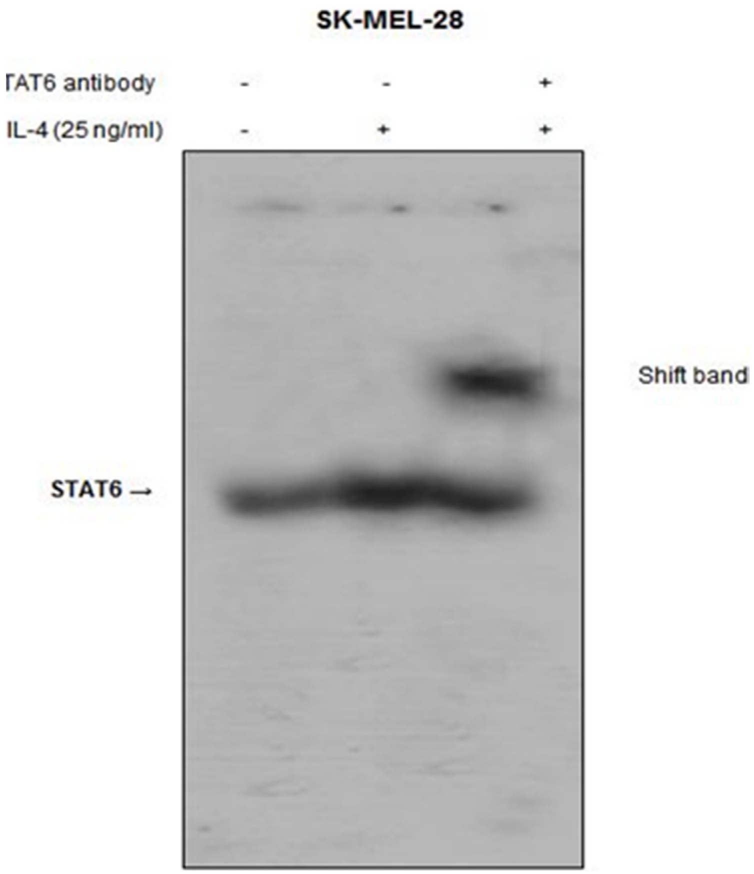


**Supplementary Figure S2: Effect of rhIL-4 on STAT6 activation in melanoma cells.** Supershift assay was performed on SK-MEL-28 melanoma cells, and a small volume of STAT6 antibody (1 µl) was added to the binding mix, and incubated at 37°C for 30 min before loading. The present results are representative for three experiments.

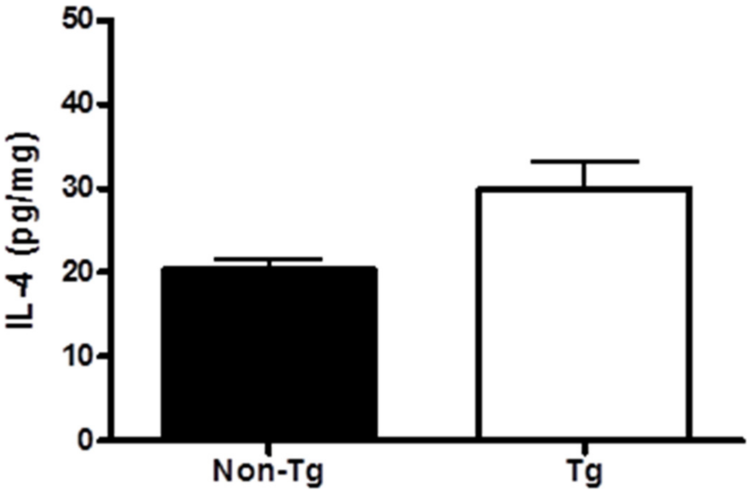


**Supplementar Figure S3: The levels of IL-4 in blood.** Differences in cytokine levels of IL-4 in Non-Tg mice and Luc/IL-4/CNS-1 mice. Results are presented as the mean ± SEM from 8 mice.
